# Editorial: New approaches to understand domestic violence and reduce its prevalence

**DOI:** 10.3389/fpsyg.2023.1120345

**Published:** 2023-01-26

**Authors:** Cristina Gonzalez-Liencres, Sofia Seinfeld, Juha Holma, Berta Vall

**Affiliations:** ^1^Max Planck School of Cognition, Leipzig, Germany; ^2^Image Processing and Multimedia Technology Center, Universitat Politècnica de Catalunya-Barcelona Tech, Barcelona, Spain; ^3^Department of Psychology, Faculty of Education and Psychology, University of Jyväskylä, Jyväskylä, Finland; ^4^Faculty of Psychology, Education and Sports Sciences, Blanquerna, Ramon Llull University, Barcelona, Spain

**Keywords:** intimate partner violence, intra-family violence, recidivism, rehabilitation program, virtual reality

Domestic violence is a major global problem encompassing distinct types of violence such as intimate partner violence (IPV), violence toward children and intra-family violence. The WHO estimates that about 30% of women worldwide have experienced physical and/or sexual intimate partner violence or non-partner sexual violence during their lifetime, mostly in the form of intimate partner violence (World Health Organization, 2021). Children can also be victims through witnessing IPV in their parents or even by experiencing direct violence against them.^1^ This Research Topic presents seven articles on this topic: four original research articles, two reviews, and one perspective. The focus of the articles ranges from the characteristics and recidivism of perpetrators, to the potential of different kinds of interventions for offenders and victims, to the impact of intimate partner violence on the victims and on their children.

Numerous countries have developed programs to reduce the recidivism of IPV perpetrators by focusing on different treatment options, even though no truly effective method has been found that works for all types of offenders (Eckhardt et al., 2013). Individualized approaches might thus be a promising option. Compatible with this idea are the findings from Askeland et al., who observed that men who voluntarily received individual psychotherapy for having committed violence against their female partner decreased their self-reported violence—partially confirmed by reports from the affected partners—from pre-treatment to post-treatment to 1.5 years after treatment. In addition, they found that more individual psychotherapy sessions decreased the likelihood of using physical violence against their partners and that clinical distress mostly declined throughout the course of psychotherapy.

Two of the articles review another type of potential approach to treat IPV offenders: the use of immersive virtual reality (VR) to enable male offenders to be in the body of a victim. In other words, the offender enters a virtual environment in which he sees his own body as that of a woman who then experiences a scene of domestic violence exerted by a man (her partner). Barnes et al. consider in particular the use of this VR tool in the context of a prison environment in which the users have been convicted. The authors defend that this VR paradigm can be integrated into rehabilitation programs in prisons and probation as it has the potential to enhance empathy and reduce violent behavior, but they also underline how several factors such as cognitive skills and stress levels can alter the efficacy. A more neuroscientific perspective is provided in the review by Johnston et al., which examines not only the behavioral outcomes of men with and without a history of violence after being exposed to the VR experience, but also the brain mechanisms underlying these effects. The authors consider how being embodied in a victim of IPV in VR—in other words, experiencing the VR scene from the first-person perspective of the victim—reduces bias, improves the recognition of emotions and diminishes violent behaviors, and they discuss the potential of VR not only for rehabilitation purposes but also for the prevention of IPV.

Rehabilitation programs are thus currently being experimentally tested and in some cases already implemented in some parts of the world. Each country deals with IPV offenders in different ways though. In Portugal, where the lifetime prevalence of physical and/or sexual IPV against women amounts to 19% (European Union Agency for Fundamental Rights, 2014), there is an option in the criminal justice system to implement what is called the Portuguese Provisional Suspension of Criminal Proceedings (PSCP), which is an alternative that proposes a consensual solution in which the concerned parties including the victim are involved in the process with the purpose of reducing the recidivism of IPV perpetrators. In their study, Vieira-Pinto et al. report that the rate of PSCP implementation is quite low (17%), which the authors defend could be due to several reasons including the victim's unawareness of this legal option, the offender's refusal to take part, or the decision by the Public Prosecution Service to deny this alternative in particular instances in which the probability of success is perceptibly low. The authors did not find evidence that PSCP decreases new re-entries into the criminal justice system within the 96 months following the first police report and warrant a deeper look into this.

Two of the abovementioned studies (Askeland et al. and Vieira-Pinto et al.) considered alcohol use as a factor that could affect changes in violence, clinical distress of the offenders or re-entry into the criminal justice system, but they did not find any significant effect. Alcohol use/abuse has indeed been often studied in the context of IPV but there is some divergence in the reported findings although alcohol seems to be aggravating (Foran and O'Leary, 2008). Sontate et al. more specifically examine here the relationship between alcohol, aggression and violence. In their review, the authors discuss the sociopsychological and neuroscientific underpinnings of alcohol use in the context of aggression and domestic violence and consider how numerous factors including comorbidity with other psychological disorders, genetic predisposition, environmental triggers and individual differences such as gender, among others, modulate this relationship. They highlight the importance of educating the public and paying attention to high-risk adolescents and parental roles with the objective of reducing alcohol intoxication-induced aggression.

Whereas, programs for perpetrators are relatively new and do not exist everywhere, most countries have developed treatments and interventions for victims of domestic violence (O'Campo et al., 2011). This is not surprising given the consequences on the victims' lives and those around them. In their original research paper, Karakurt et al. look in particular at traumatic brain injury (TBI) and mental health of female victims in a case-series study with three women with a history of IPV without any experience of concussive head trauma, and three women with a history of IPV with concussive head trauma. The authors found that the groups differed in the resting-state connectivity, in relationship dynamics and in mental health symptoms, and emphasize the need for accurate and early characterization of TBI in IPV victims in order to guide treatment and ameliorate the health-related consequences.

Direct victims of IPV are not the only victims: children whose parents experience domestic violence are also at higher risk of developing psychological issues. Glaus et al. explore children's psychopathology in households with domestic violence toward the mother and its relationship with maternal post-traumatic stress disorder (PTSD) owing to IPV. This longitudinal study shows that lifetime exposure to violence exacerbates the development of psychopathology in children, as does the fact that their mother has experienced IPV.

In conclusion, these studies touch upon the Research Topic of domestic violence from separate perspectives and stress the need to adequately identify and treat the victims and other affected members of the household, the need to find effective intervention programs for the batterers, and the importance of having legal alternatives that benefit all parts and dissuade this global issue.

## Author contributions

CG-L wrote the first draft. All authors contributed to the final version of this Editorial.

## Footnotes

^1^ https://www.who.int/news-room/fact-sheets/detail/violence-against-children
